# Supplementary material for: Reconstruction of the lymphatic system by transplantation of a centrifuge-based bioengineered lymphatic tissue
Source: Nat Commun. 2025 Nov 19;16:10154. doi: 10.1038/s41467-025-65121-3 (PMC12630873; doi:10.1038/s41467-025-65121-3)
Supplement: Supplementary file 1 — Supplementary Information [file 41467_2025_65121_MOESM1_ESM.pdf]

Title

Reconstruction of the lymphatic system by transplantation of a centrifuge-based bioengineered lymphatic tissue

Authors

Shu Obana[1], Shoko Itakura[2], Mutsunori Murahashi[3], Makiya Nishikawa[2], Kosuke Kusamori[1,*]

Affiliations

[1]Laboratory of Cellular Drug Discovery and Development, Faculty of Pharmaceutical Sciences, Tokyo University of Science, 6-3-1 Niijuku, Katsushika, Tokyo 125-8585, Japan

[2]Laboratory of Biopharmaceutics, Faculty of Pharmaceutical Sciences, Tokyo University of Science, 6-3-1 Niijuku, Katsushika, Tokyo 125-8585, Japan

[3]Division of Oncology, Research Center for Medical Sciences, The Jikei University School of Medicine, Tokyo, Japan.

*Corresponding author

Kosuke Kusamori, Ph.D.

Tel./Fax.: +81-3-5876-1747 (Ext. 6512)

E-mail address: kusamori@rs.tus.ac.jp

Supplementary Figures

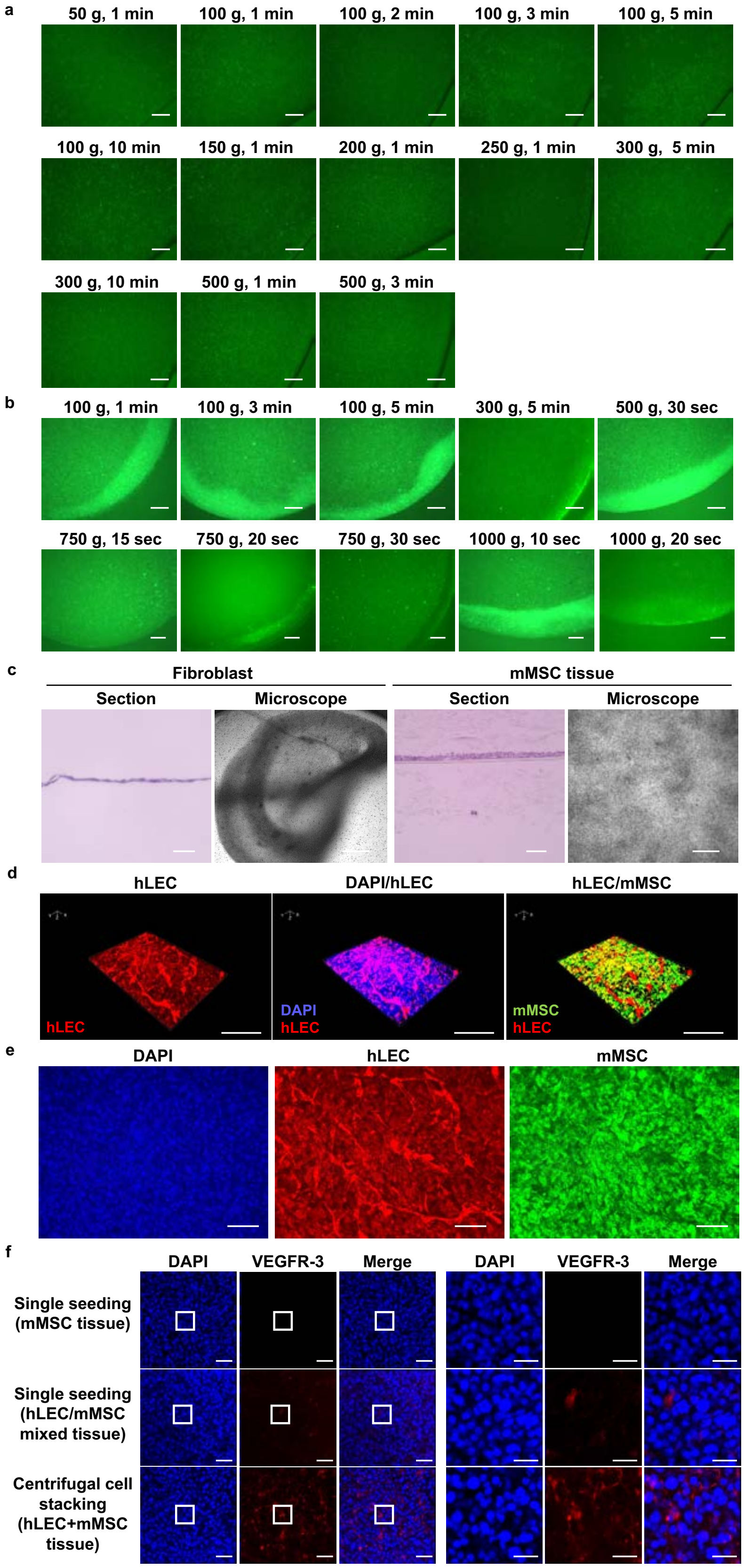

Supplementary Fig. 1. | Optimization of centrifugation conditions for the preparation of CeLyTs.

a, b, Fluorescence microscopic observation of mMSCs/GFP after centrifugation under various conditions. (a) Seeding once and (b) twice (day 1) of > 3 independent experiments. Scale bars, 500 µm. **c,** Paraffin-sections and microscopic images of fibroblast cells and mMSC tissue (day 5) of > 3 independent experiments. Scale bars, 50 µm. **d, e,** Typical 3D images (d) and fluorescence microscopic images (e) of an hLEC+mMSC tissue. Blue, DAPI; Green, CellTracker™ Green-labeled mMSC; Red, CellTracker™ Orange-labeled hLECs. Scale bars, 100 µm. **f,** Immunofluorescence images of a lymphatic endothelial cell marker VEGFR-3 in mMSC tissues (by single seeding without centrifugation), hLEC/mMSC mixed tissue (by single seeding without centrifugation), and hLEC+mMSC tissue (by centrifugal cell stacking technique). Blue, DAPI; Red, VEGFR-3. Scale bars, 100 µm (left panel) and 50 µm (right panel).

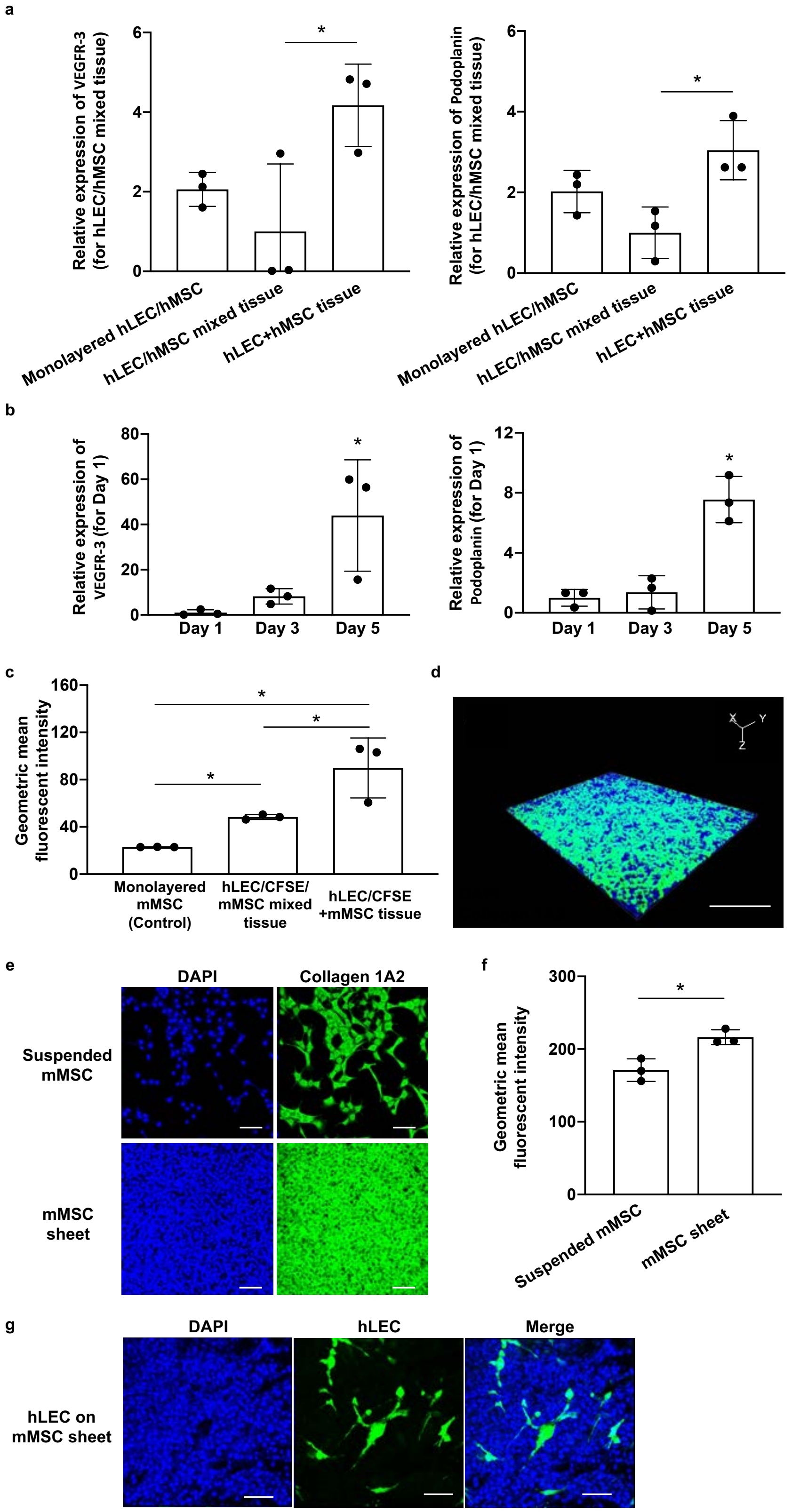
a
Relative expression of VEGFR-3 (for hLEC/hMSC mixed tissue)
Relative expression of Podoplanin (for hLEC/hMSC mixed tissue)
Monolayered hLEC/hMSC
hLEC/hMSC mixed tissue
hLEC+hMSC tissue
b
Relative expression of VEGFR-3 (for Day 1)
Relative expression of Podoplanin (for Day 1)
Day 1
Day 3
Day 5
c
Geometric mean fluorescent intensity
Monolayered mMSC (Control)
hLEC/CFSE/ mMSC mixed tissue
hLEC/CFSE +mMSC tissue
d
e
DAPI
Collagen 1A2
Suspended mMSC
mMSC sheet
f
Geometric mean fluorescent intensity
Suspended mMSC
mMSC sheet
g
DAPI
hLEC
Merge
hLEC on mMSC sheet

Supplementary Fig. 2. | Optimization of the centrifugation conditions for the preparation of CeLyTs.

a, The relative mRNA expressions of VEGF-R3 and Podoplanin in monolayered hLEC/mMSC and hLEC+mMSC tissues for hLEC/mMSC mixed tissues. Data represent the mean ± standard deviation from 3 independent experiments, and P-values were determined by two-sided Dunnett's test. *$P < 0.05$ was considered statistically significant. **b,** The relative mRNA expressions of VEGF-R3 and Podoplanin on days 3 and 5 compared relative to day 1. Data represent the mean ± standard deviation from 3 independent experiments, and P-values were determined by two-sided Dunnett's test. *$P < 0.05$ was considered significant. **c,** Flow cytometric analysis of the viability of hLECs in hLEC/CFSE/mMSC mixed tissues and hLEC/CFSE+hMSC tissues (day 5). Monolayered mMSCs were used as a negative control. Data represent the mean ± standard deviation from 3 independent experiments, and P-values were determined by the two-sided Tukey-Kramer test. *$P < 0.05$ was considered statistically significant. **d,** Typical 3D image of an mMSC+hLEC tissue. Blue, DAPI; Green, Collagen 1A2. Scale bar, 100 μm. **e, f,** Fluorescence microscopic observations (e) and flow cytometric analysis (f) of suspended mMSCs and mMSC sheets. Blue, DAPI; Green, Collagen 1A2. Scale bars, 100 μm. Data represent the mean ± standard deviation from 3 independent experiments, and P-values were determined by two-tailed Student's *t*-test. *$P < 0.05$ was considered statistically significant. **g,** Immunofluorescence images of the lymphatic network of hLEC seeded on mMSC sheets. Blue, DAPI; Green, Collagen 1A2. Scale bars, 100 μm.

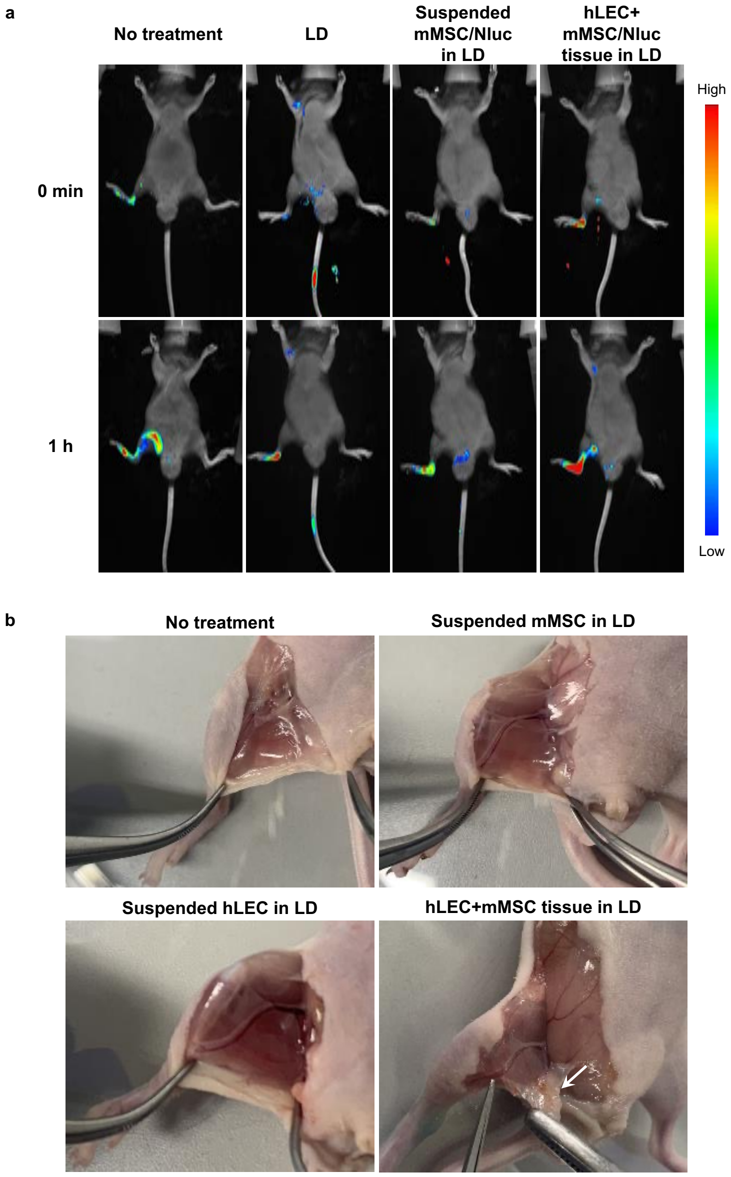

Supplementary Fig. 3. | Preparation of LD mice.

a, *In vivo* fluorescence imaging 0 min and 1 h after ICG injection into the footpad of the right limbs of normal mice (no treatment), LD mice, and LD mice 14 days after suspended mMSC/Nluc or hLEC+mMSC/Nluc tissue transplantation. **b**, Typical images of the suspended cells or CeLyTs transplantation site in a right lower limb of an LD mouse (day 21). Arrow, lymph node-like structure.

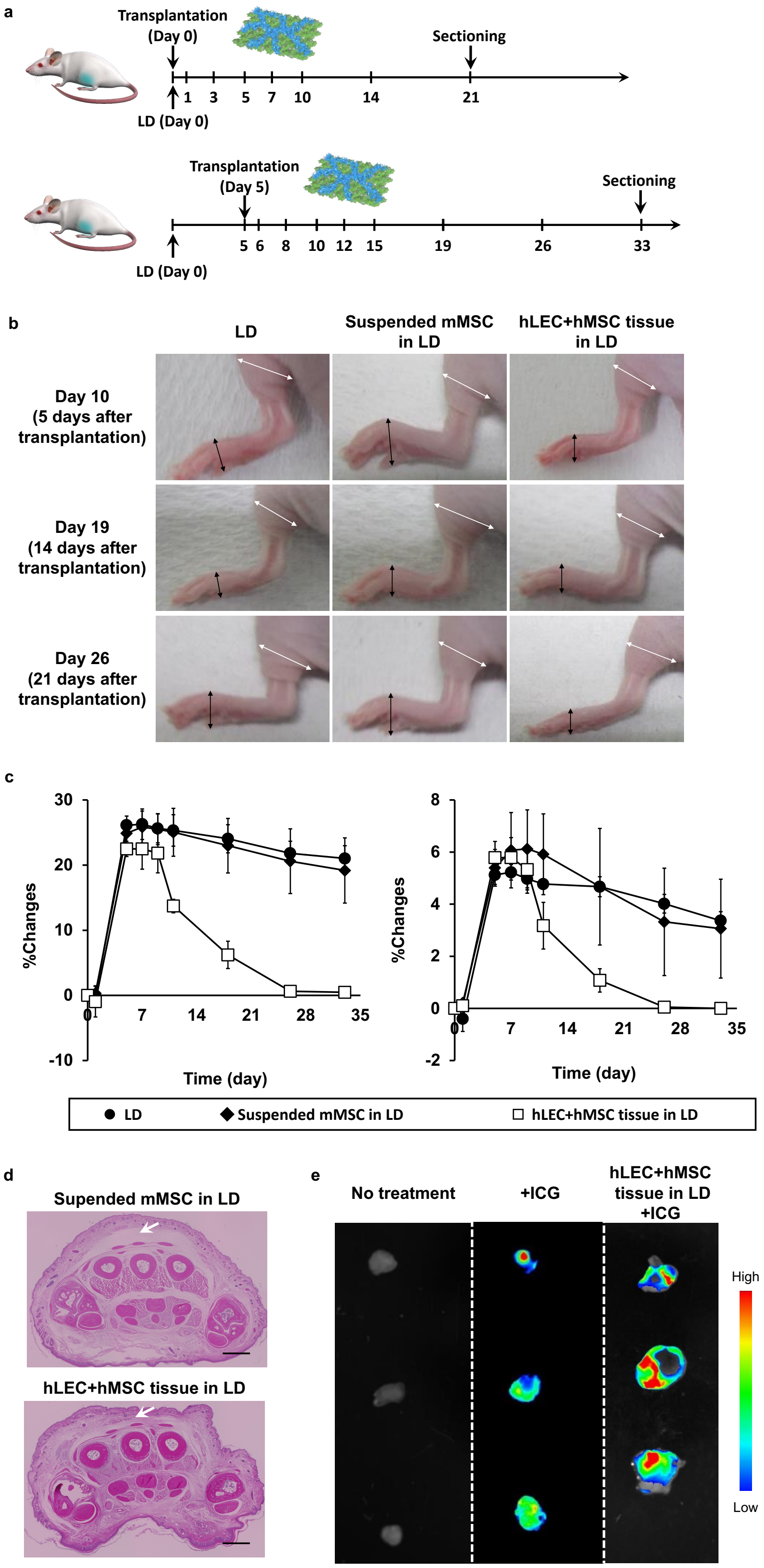
a
Transplantation
(Day 0)
Sectioning
1 3 5 7 10 14 21
LD (Day 0)
Transplantation
(Day 5)
Sectioning
5 6 8 10 12 15 19 26 33
LD (Day 0)
b
LD
Suspended mMSC in LD
hLEC+hMSC tissue in LD
Day 10 (5 days after transplantation)
Day 19 (14 days after transplantation)
Day 26 (21 days after transplantation)
c
%Changes
Time (day)
● LD
◆ Suspended mMSC in LD
□ hLEC+hMSC tissue in LD
d
Supended mMSC in LD
hLEC+hMSC tissue in LD
e
No treatment
+ICG
hLEC+hMSC tissue in LD +ICG
High
Low

Supplementary Fig. 4. | Therapeutic effect of CeLyTs on LD mice.
a, Experimental scheme to evaluate the therapeutic effect of CeLyTs on lymphedema in LD mice. CeLyTs were transplanted to LD mice immediately (upper) or 5 days (lower) after LD. **b,** Typical images of right lower limbs from suspended mMSCs- and hLEC+hMSC tissue-transplanted LD mice. Arrows indicate the measurement site (white, legs; black, paws) in the lower limbs for the evaluation of edema. **c,** Change in the size of paws (left) and legs (right) in suspended mMSCs- and hLEC+hMSC tissue-transplanted LD mice. The thicknesses of paws and legs were measured and the change rate for day 0 was calculated. Data represent the mean ± standard deviation from 3 independent experiments. **d**, Paraffin-section images of paws from suspended mMSCs- and hLEC+hMSC tissue-transplanted LD mice (day 26). Arrows indicate the interstitial fluid. Scale bars, 500 µm. **e**, *Ex vivo* imaging of lymph nodes or lymph node-like structures after ICG administration to normal mice (+ICG) and hLEC+hMSC tissue-transplanted LD mice (day 26). Supplementary Fig. 4a was created by Science Graphics Inc. (Kyoto, Japan) under a commissioned agreement. All rights have been transferred to the corresponding author.

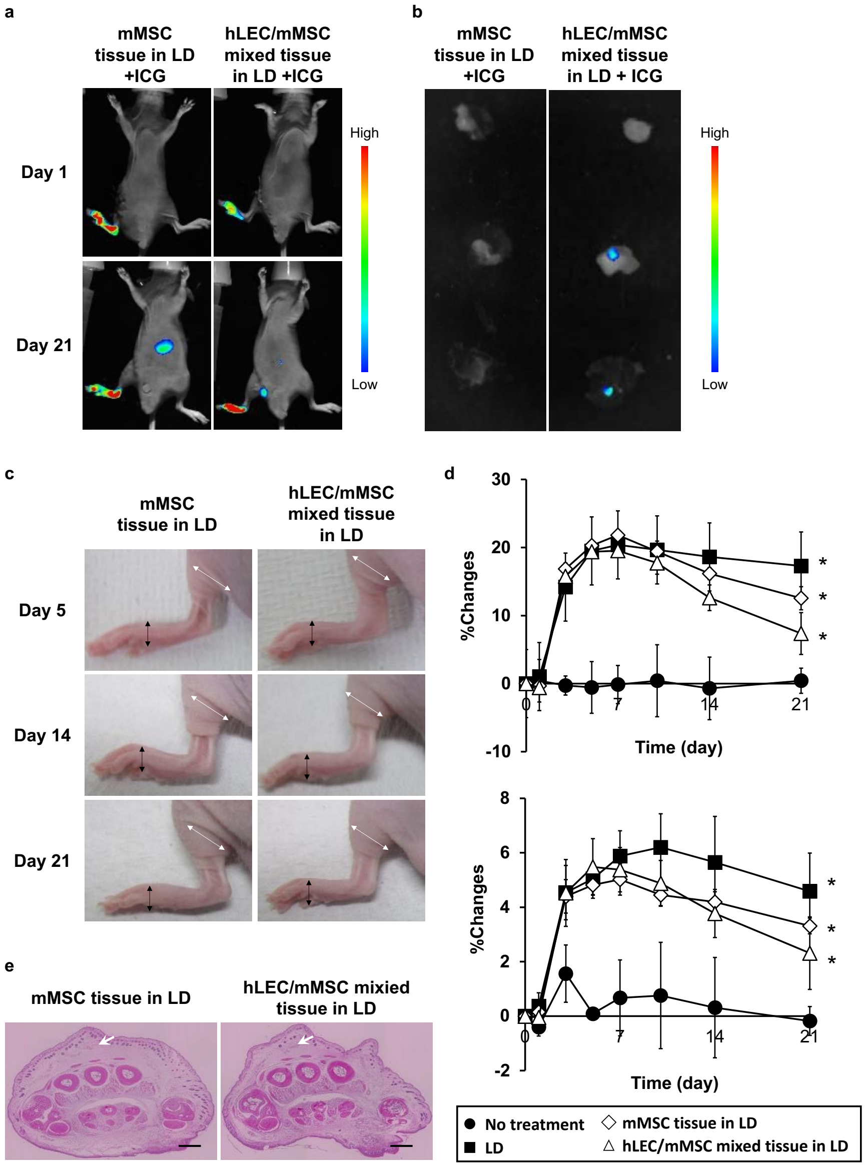

Supplementary Fig. 5. | Therapeutic effect of transplantation of other tissues on LD mice.

a, *In vivo* fluorescence imaging after an injection of ICG to the footpad of right lower limbs of LD mice 1 and 21 days after the transplantation of mMSC tissues and hLEC/mMSC mixed tissues. **b,** *Ex vivo* imaging of lymph nodes or lymph node-like structures after ICG administration in mMSC tissues and hLEC/mMSC mixed tissue-transplanted LD mice (day 21). **c,** Typical images of right lower limbs of LD mice after the transplantation of mMSC tissues and hLEC/mMSC mixed tissues. Arrows indicate the measurement site (white, legs; black, paws) in the lower limbs for the evaluation of edema. **d,** Size change of paws (upper) and legs (lower) in LD mice after transplantation

of mMSC tissues and hLEC/mMSC mixed tissues. The thickness of paws and legs was measured and the change rate for day 0 was calculated. Data represent the mean ± standard deviation from 3 independent experiments, and P-values were determined by two-sided Dunnett's test. *$P < 0.05$ was considered statistically significant for the No treatment group. ns, not significant. **e**, Paraffin-section images of paws in mMSC tissue- and hLEC/mMSC mixed tissue-transplanted LD mice (day 21). Arrows indicate the interstitial fluid. Scale bars, 500 μm.

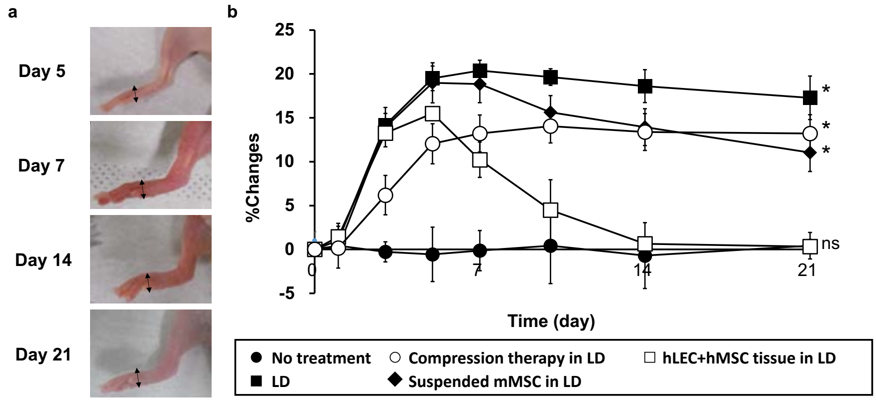

Supplementary Fig. 6. | Comparison of therapeutic effect of CeLyTs with the compression therapy as a gold standard treatment for secondary lymphedema.

a, Typical images of right lower limbs of LD mice undergoing compression therapy. Arrows indicate the measurement site (black, paws) in the lower limbs for the evaluation of edema. **b,** Change in the size of paws in compression therapy, suspended mMSC- and hLEC+hMSC tissue-transplanted LD mice. The thicknesses of the paws were measured and the change rate for day 0 was calculated. Data represent the mean ± standard deviation from 3 independent experiments, and P-values were determined by two-sided Dunnett's test. *$P < 0.05$ was considered statistically significant for the No treatment group. ns, not significant.

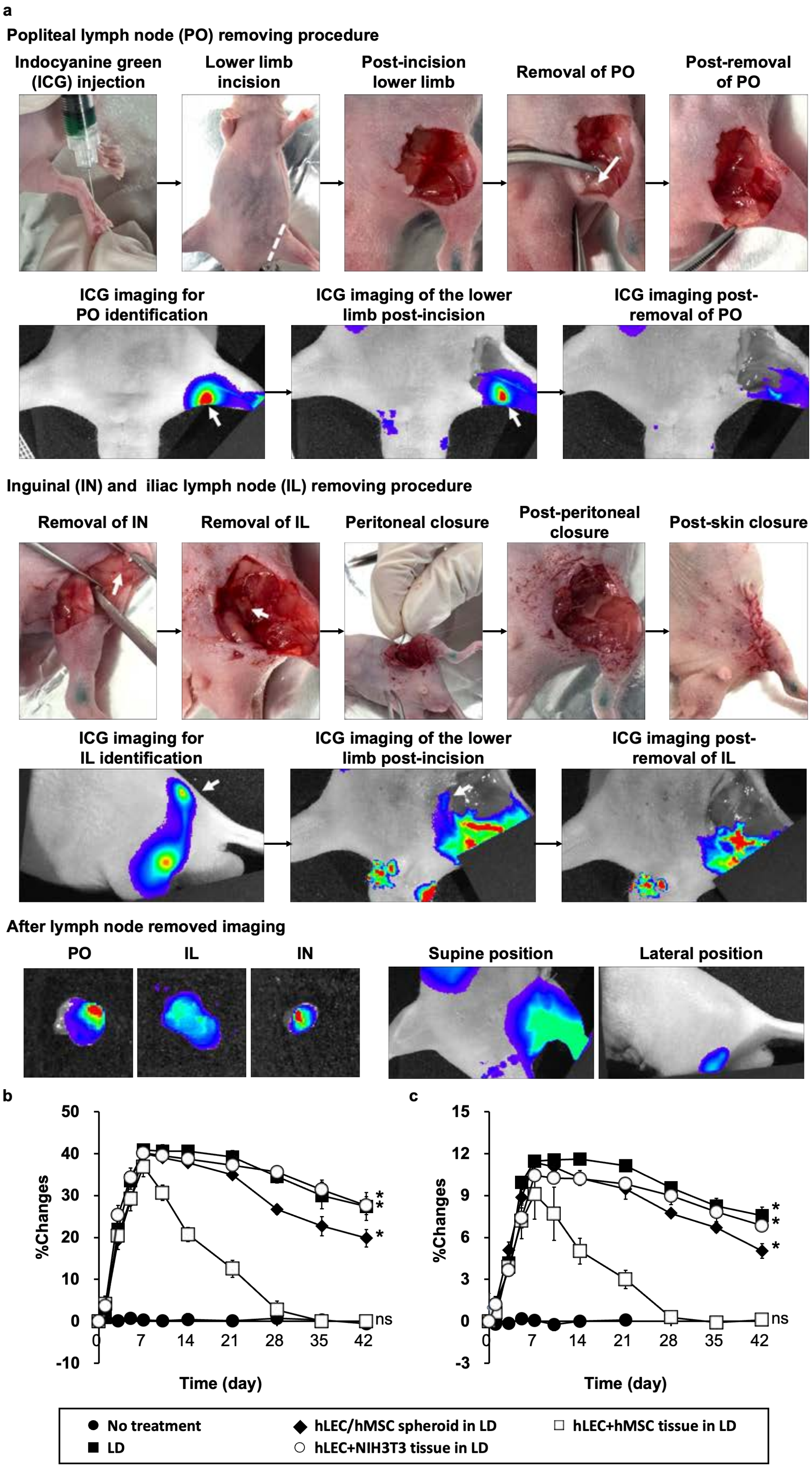
a
Popliteal lymph node (PO) removing procedure
Indocyanine green (ICG) injection
Lower limb incision
Post-incision lower limb
Removal of PO
Post-removal of PO
ICG imaging for PO identification
ICG imaging of the lower limb post-incision
ICG imaging post-removal of PO
Inguinal (IN) and iliac lymph node (IL) removing procedure
Removal of IN
Removal of IL
Peritoneal closure
Post-peritoneal closure
Post-skin closure
ICG imaging for IL identification
ICG imaging of the lower limb post-incision
ICG imaging post-removal of IL
After lymph node removed imaging
PO
IL
IN
Supine position
Lateral position
b
%Changes
Time (day)
ns
c
%Changes
Time (day)
ns
No treatment
LD
hLEC/hMSC spheroid in LD
hLEC+NIH3T3 tissue in LD
hLEC+hMSC tissue in LD

Supplementary Fig. 7. | Therapeutic effect of bioengineered tissues with lymphatic network in chronic lymphedema model mice.

a, Surgical procedures for popliteal (PO), inguinal (IN), and iliac (IL) lymph node removal and corresponding Indocyanine green (ICG) imaging. For PO removal, ICG was injected into the footpad, and the lower limb was incised along the white dotted line (~1.5 cm). Guided by ICG imaging, the PO was identified and excised. Fluorescence in the PO region disappeared immediately after removal. Arrows: PO. For IN and IL removal, the IN, located at the confluence of three major vessels, was excised first. Arrows: IN. The peritoneum was then incised to expose the IL, which was resected under real-time ICG imaging guidance. Arrows: IL. The peritoneum was closed with nylon sutures, followed by skin closure. After lymph node removal, *ex vivo* imaging confirmed the removal of PO, IN, and IL. *In vivo* imaging immediately after removal demonstrated dispersal of ICG signal, consistent with interruption of lymphatic flow. **b, c,** Size change of paws (b) and legs (c) in chronic lymphedema model mice after transplantation of hLEC/hMSC spheroids, hLEC+NIH3T3 tissue, and hLEC+hMSC tissue. The thickness of paws and legs was measured for 42 days and the change rate for day 0 was calculated. Data represent the mean ± standard deviation from 3 independent experiments, and P-values were determined by two-sided Dunnett's test. *$P < 0.05$ was considered statistically significant for the No treatment group. ns, not significant.

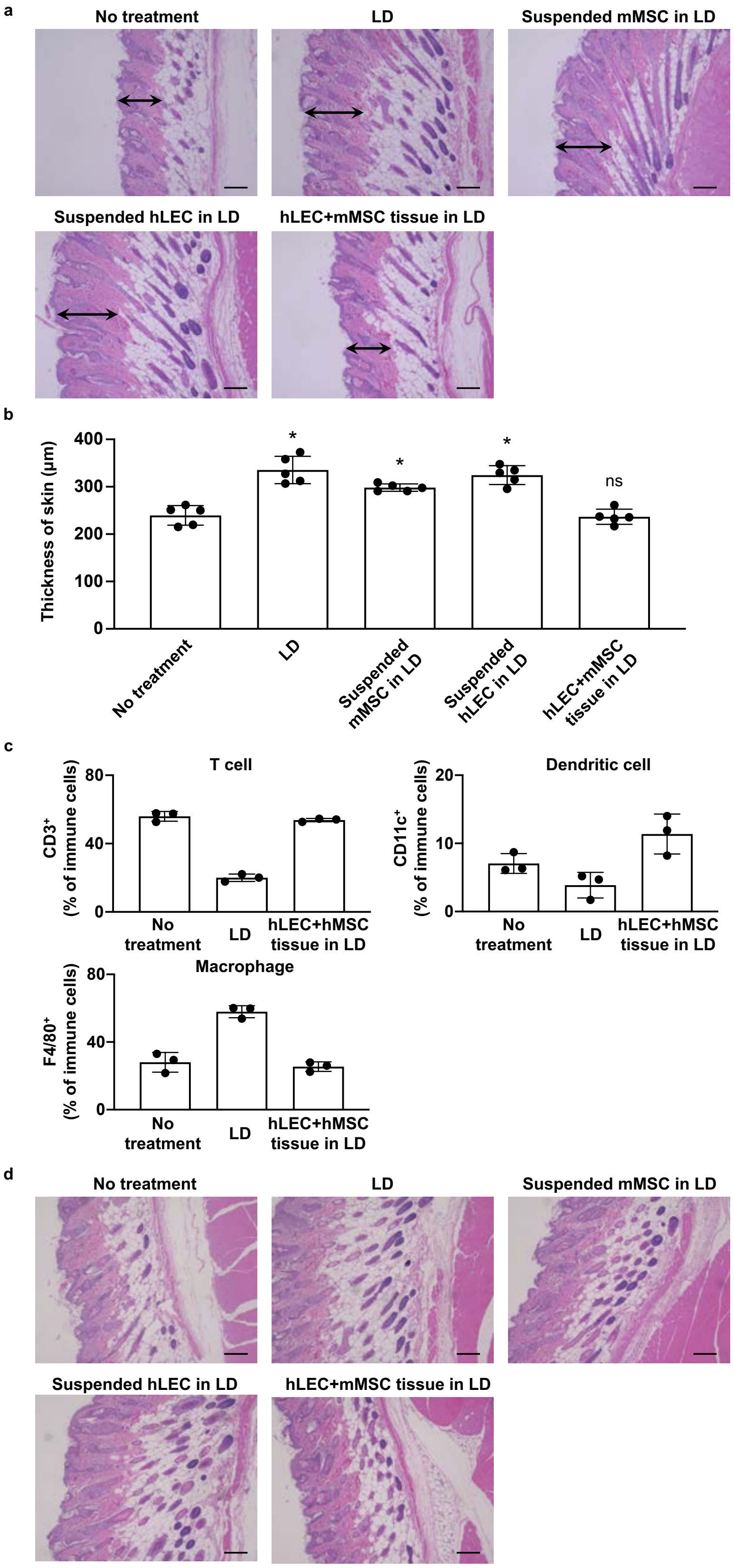
a
No treatment
LD
Suspended mMSC in LD
Suspended hLEC in LD
hLEC+mMSC tissue in LD
b
Thickness of skin (μm)
400
300
200
100
0
*
*
*
ns
No treatment
LD
Suspended mMSC in LD
Suspended hLEC in LD
hLEC+mMSC tissue in LD
c
T cell
CD3⁺ (% of immune cells)
80
40
0
No treatment
LD
hLEC+hMSC tissue in LD
Dendritic cell
CD11c⁺ (% of immune cells)
20
10
0
No treatment
LD
hLEC+hMSC tissue in LD
Macrophage
F4/80⁺ (% of immune cells)
80
40
0
No treatment
LD
hLEC+hMSC tissue in LD
d
No treatment
LD
Suspended mMSC in LD
Suspended hLEC in LD
hLEC+mMSC tissue in LD

Supplementary Fig. 8. | Suppressive effect on lymphedema hallmarks after transplantation of bioengineered tissues with lymphatic network in LD.

a, Paraffin-section images of skin of Suspended cells- and bioengineered tissue-transplanted LD mice (day 21). Arrows, measurement sites for the thickness of skin. Scale bars, 500 μm. **b,** The thicknesses of skin were measured from five sites. Data represent the mean ± standard deviation from 5 sites in the sections, and P-values were determined by two-sided Dunnett's test. *P < 0.05 was considered statistically significant for No treatment. ns, not significant. **c,** Proportion of various immune cell populations in the dermis of hLEC+hMSC tissue-transplanted LD mice (day 21). $CD3^{+}$ cells, $CD11c^{+}$ cells, and $F4/80^{+}$ cells were gated by $CD45^{+}$ cells. Data represent the mean ± standard deviation from 3 independent experiments. **d,** Paraffin-section images of adipose tissue under the skin of suspended cells- and bioengineered tissue-transplanted LD mice (day 21).

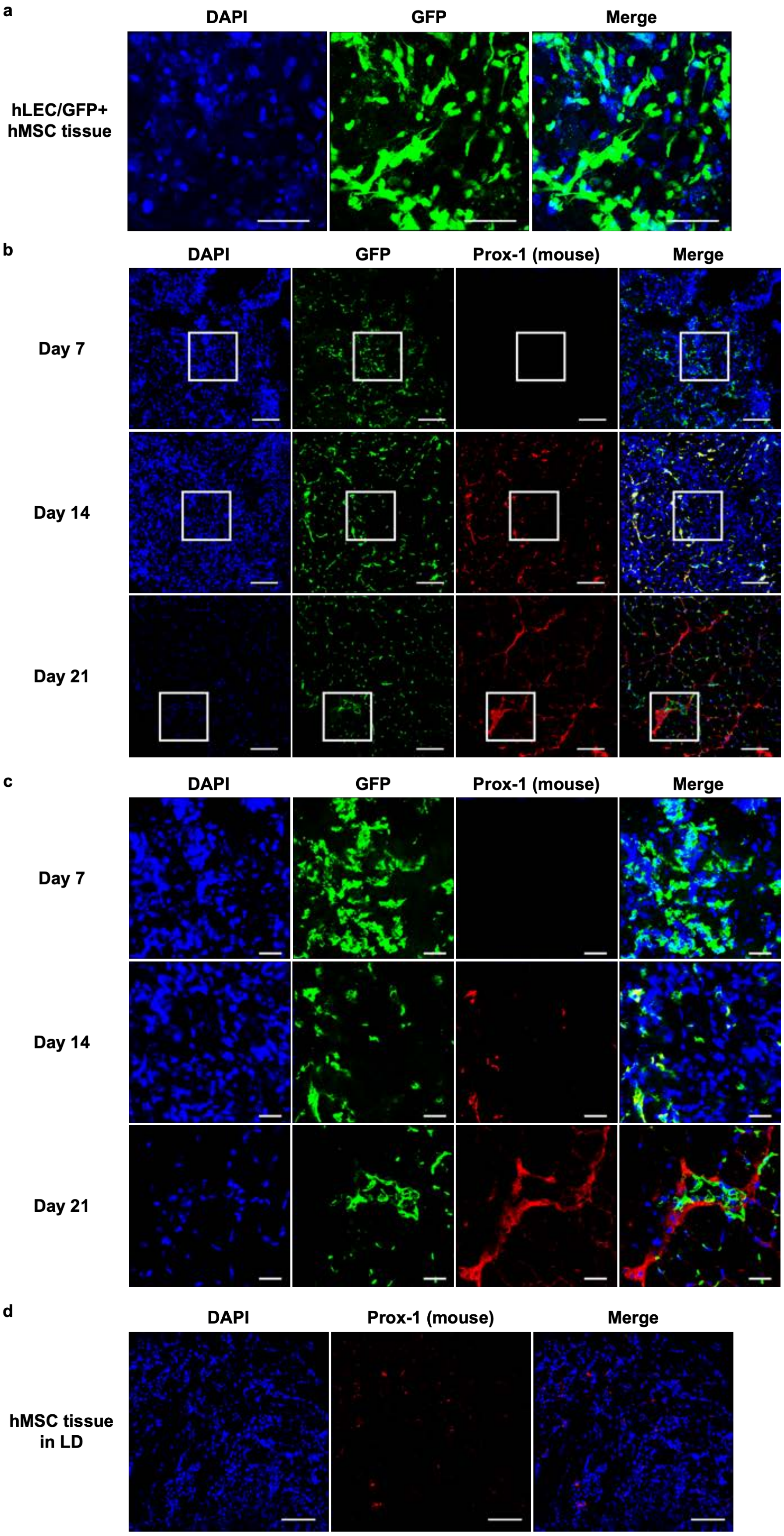
a
DAPI
GFP
Merge
hLEC/GFP+ hMSC tissue
b
DAPI
GFP
Prox-1 (mouse)
Merge
Day 7
Day 14
Day 21
c
DAPI
GFP
Prox-1 (mouse)
Merge
Day 7
Day 14
Day 21
d
DAPI
Prox-1 (mouse)
Merge
hMSC tissue in LD

Supplementary Fig. 9. | Presence of transplanted LECs and their association with host-derived LECs in CeLyTs.

a, Fluorescence images of hLEC/GFP+hMSC tissue by centrifugal cell stacking technique (day 5). Blue, DAPI; Green, GFP. Scale bars, 100 µm. **b,** Representative immunofluorescence images of mouse Prox-1 (a lymphatic endothelial cell marker) and GFP derived from hLEC/GFP in lymph node-like structures formed in hLEC/GFP+hMSC tissue-transplanted LD mice (days 7, 14, and 21). Blue, DAPI; Green, GFP; Red, mouse-reactive Prox-1. Scale bars, 100 µm. **c,** Enlarged images in the white boxed areas of **b**. Blue, DAPI; Green, GFP; Red, mouse-reactive Prox-1. Scale bars, 30 µm. **d,** Representative immunofluorescence images of mouse Prox-1 (a lymphatic endothelial cell marker) in hMSC tissue-transplanted LD mice (day 21). Blue, DAPI; Red, mouse-reactive Prox-1. Scale bars, 100 µm.

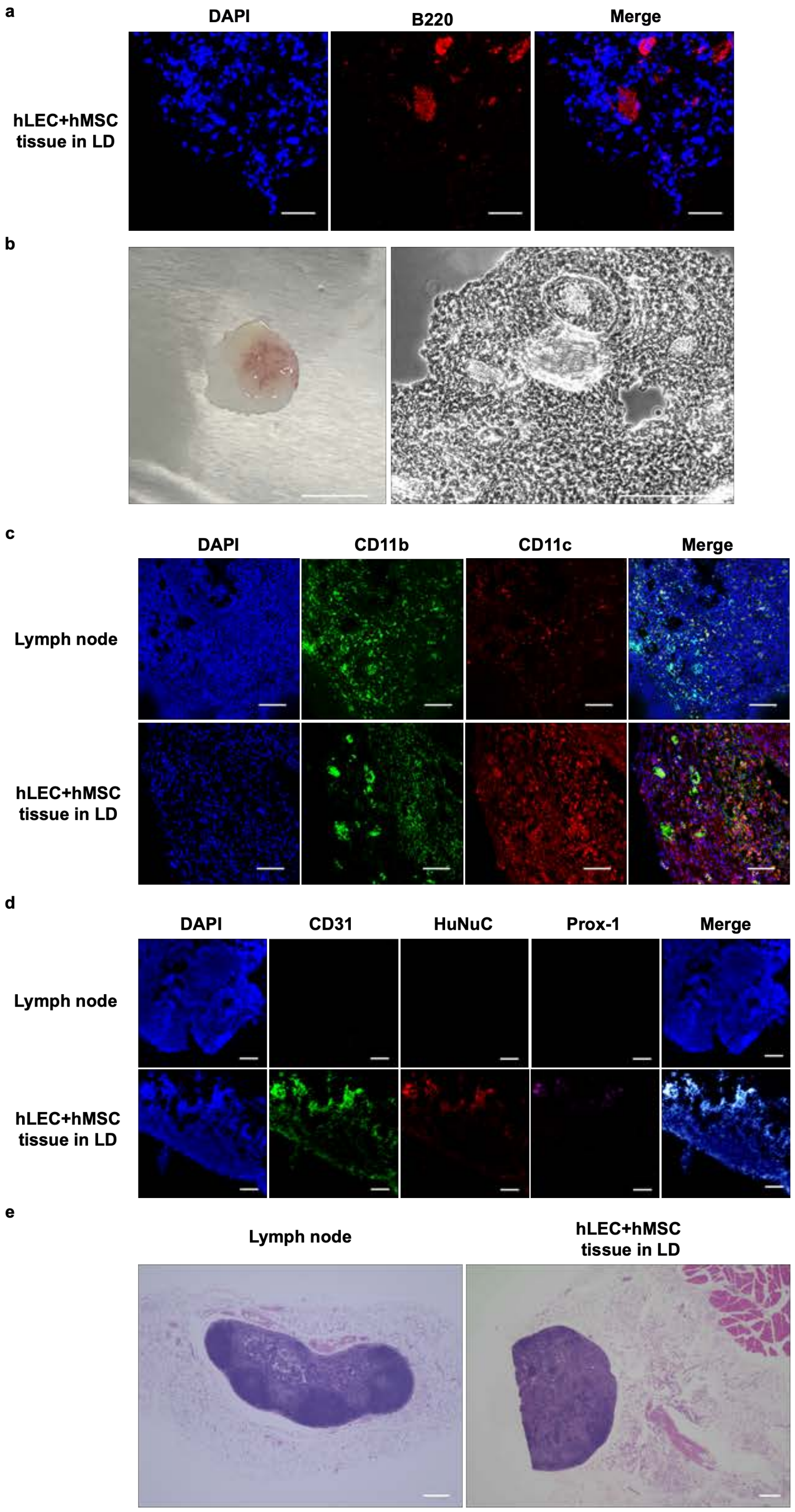
a
DAPI
B220
Merge
hLEC+hMSC tissue in LD
b
c
DAPI
CD11b
CD11c
Merge
Lymph node
hLEC+hMSC tissue in LD
d
DAPI
CD31
HuNuC
Prox-1
Merge
Lymph node
hLEC+hMSC tissue in LD
e
Lymph node
hLEC+hMSC tissue in LD

Supplementary Fig. 10. | Analysis of lymph node-like structures after transplantation of CeLyTs.

a, Representative immunofluorescence images of B cell follicle-like structures in a lymph node-like structure formed in hLEC+hMSC tissue-transplanted LD mice (day 21). Blue, DAPI; Red, B220. Scale bars, 100 μm. **b,** Appearance and frozen section image of the lymph node-like structure formed in hLEC+hMSC tissue-transplanted LD mice (day 21). **c,** Representative immunofluorescence images of CD11b (a macrophage marker) and CD11c (a dendritic cell marker) in lymph nodes or lymph node-like structures formed in hLEC+hMSC tissue-transplanted LD mice (day 21) of > 3 independent experiments. Blue, DAPI; Green, CD11b; Red, CD11c. Scale bars, 100 μm. **d,** Representative immunofluorescence images of human CD31 (an endothelial cell marker), human Prox-1 (a lymphatic endothelial cell marker), and HuNuC (Human nuclei) in lymph nodes or lymph node-like structures formed in hLEC+hMSC tissue-transplanted LD mice (day 21) of > 3 independent experiments. Blue, DAPI; Green, human-reactive CD31; Red, HuNuC: Magenta, human-reactive Prox-1. Scale bars, 100 μm. **e,** Paraffin-section images of a lymph node and lymph node-like structure formed in hLEC+hMSC tissue-transplanted LD mice (day 21). Scale bars, 500 μm.

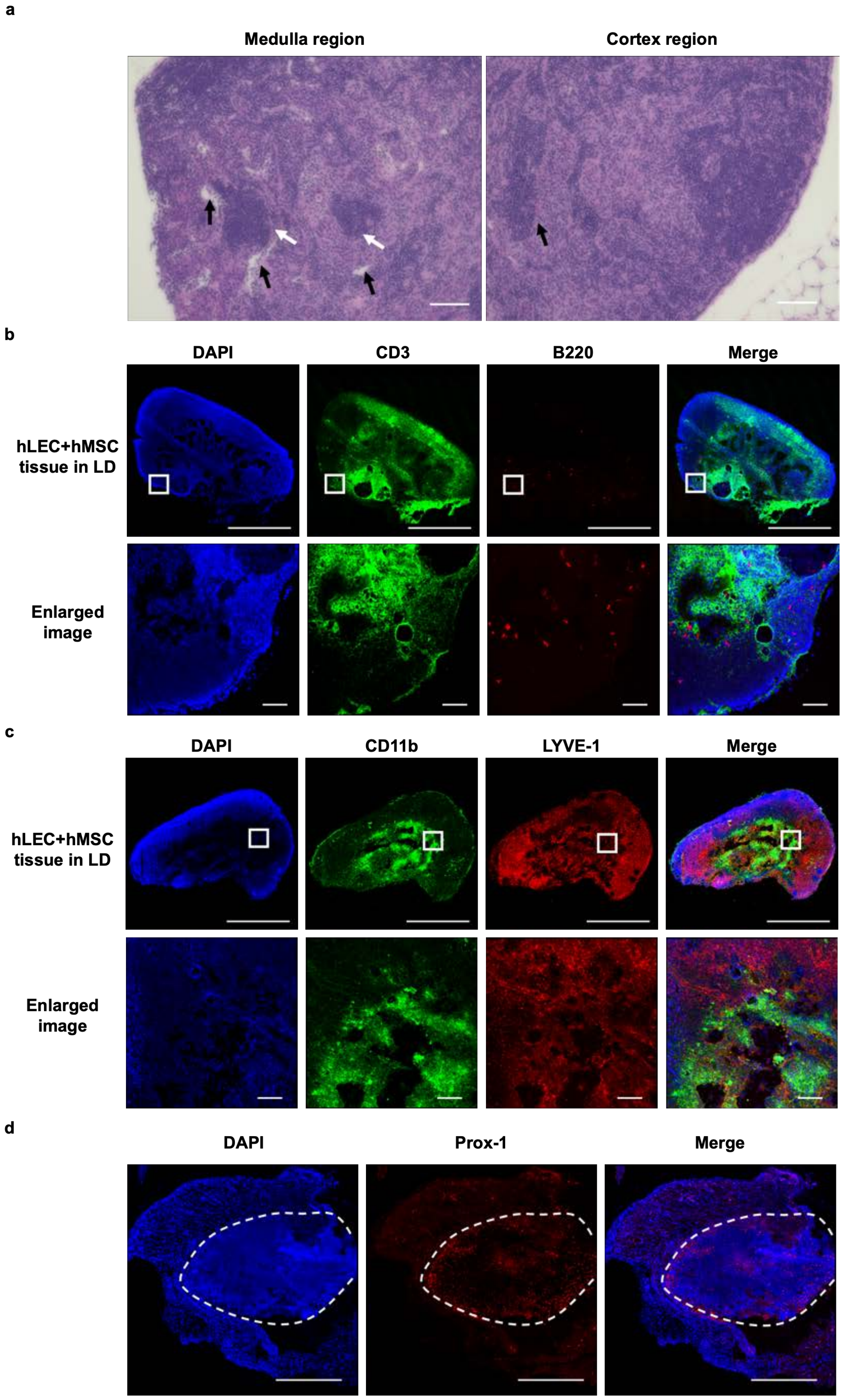
a
Medulla region
Cortex region
b
DAPI
CD3
B220
Merge
hLEC+hMSC tissue in LD
Enlarged image
c
DAPI
CD11b
LYVE-1
Merge
hLEC+hMSC tissue in LD
Enlarged image
d
DAPI
Prox-1
Merge

216

Supplementary Fig. 11. | Structural analysis of lymph node-like structures after transplantation of CeLyTs.

a, Paraffin-section images of a medulla and a cortex region in a lymph node-like structure formed in hLEC+hMSC tissue-transplanted LD mice (day 21). Scale bars, 200 μm. Black arrows, sinuses; white arrows, follicles. **b**, Representative immunofluorescence staining for CD3 and B220 in a lymph node-like structure formed in hLEC+hMSC tissue-transplanted LD mice (day 21) of > 3 independent experiments. Blue, DAPI; Green, CD3; Red, B220. Scale bars, 1 mm (upper panel) and 200 μm (lower panel). **c**, Representative immunofluorescence staining for CD11b and LYVE-1 in a lymph node-like structure formed in hLEC+hMSC tissue-transplanted LD mice (day 21) of > 3 independent experiments. Blue, DAPI; Green, CD11b; Red, LYVE-1. Scale bars, 1 mm (upper panel) and 200 μm (lower panel). **d**, Representative immunofluorescence staining for Prox-1 in a lymph node-like structure formed in hLEC+hMSC tissue-transplanted LD mice with the surrounding host muscle tissue (day 21). Blue, DAPI; Red, Prox-1. Scale bars, 1 mm. Within the white dot circle: lymph node-like structure.

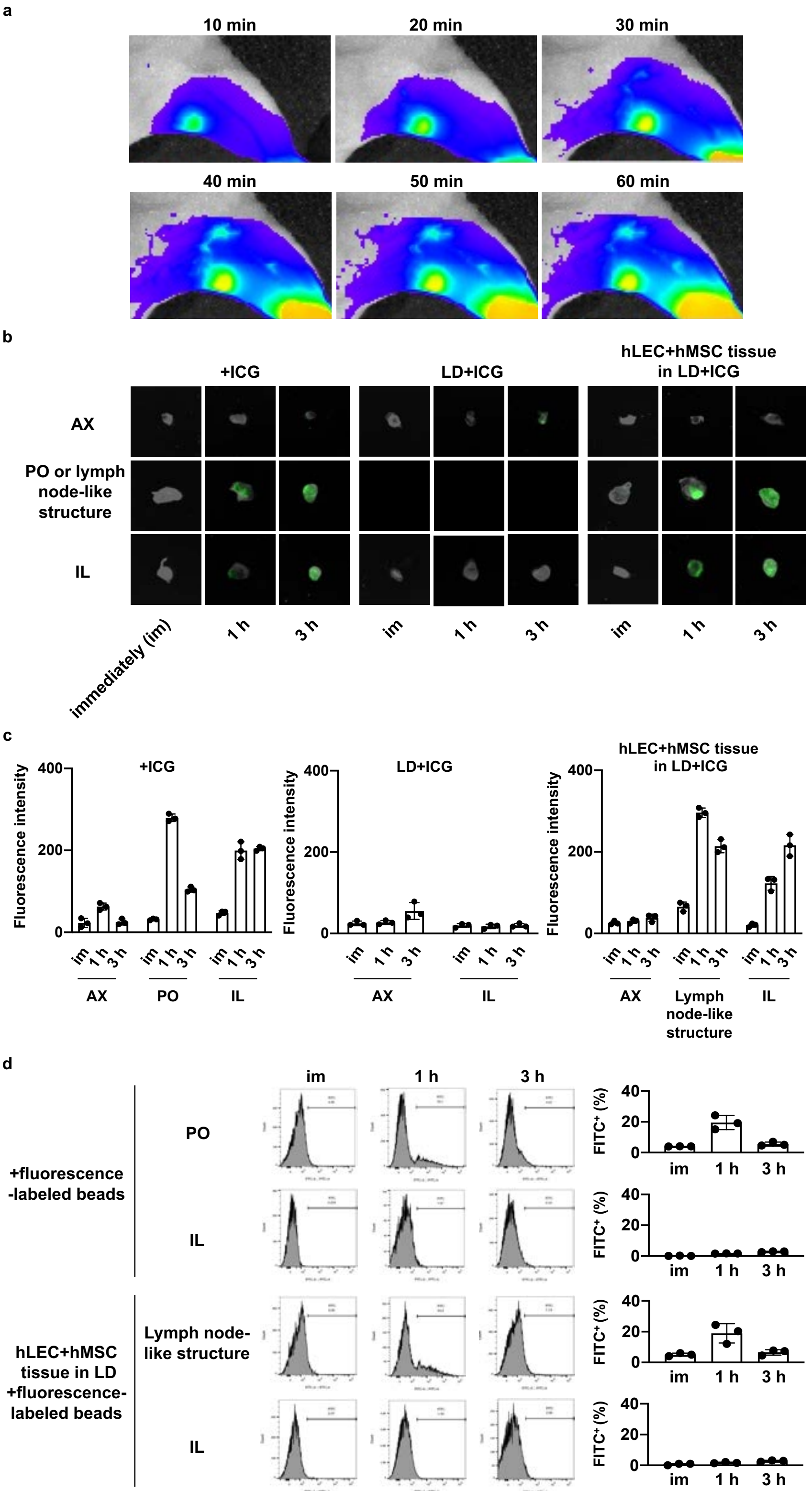
a
10 min
20 min
30 min
40 min
50 min
60 min
b
+ICG
LD+ICG
hLEC+hMSC tissue
in LD+ICG
AX
PO or lymph
node-like
structure
IL
immediately (im)
1 h
3 h
im
1 h
3 h
im
1 h
3 h
c
+ICG
LD+ICG
hLEC+hMSC tissue
in LD+ICG
Fluorescence intensity
400
200
0
im
1 h
3 h
AX
PO
IL
Lymph
node-like
structure
d
im
1 h
3 h
PO
IL
+fluorescence
-labeled beads
Lymph node-
like structure
hLEC+hMSC
tissue in LD
+fluorescence-
labeled beads
FITC⁺ (%)
40
20
0

Supplementary Fig. 12. | Lymphatic flow in lymph node-like structure after transplantation of bioengineered tissues with lymphatic network

a, *In vivo* imaging of lymph flow in lymph node-like structures formed in hLEC+hMSC tissue-transplanted LD mice (day 21) after ICG injection. **b,** *Ex vivo* imaging of lymph nodes or lymph node-like structures immediately (im), 1 h, and 3 h after ICG administration to normal mice (+ICG), LD mice (LD+ICG), and hLEC+hMSC tissue-transplanted LD mice (day 21). **c,** Fluorescent intensity of excised and homogenized lymph nodes or lymph node-like structures immediately (im), 1 h, or 3 h after ICG administration. Data represent the mean ± standard deviation from 3 independent experiments. **d,** Flow cytometric analysis of filtration capacity monocyte population in lymph nodes or lymph node-like structures immediately (im), 1 h, and 3 h after fluorescence-labeled beads administration to normal mice (No treatment) and hLEC+hMSC tissue-transplanted LD mice (day 21). Data represent the mean ± standard deviation from 3 independent experiments. AX, Axillary lymph node; PO, Popliteal lymph node; IL, Iliac lymph node.

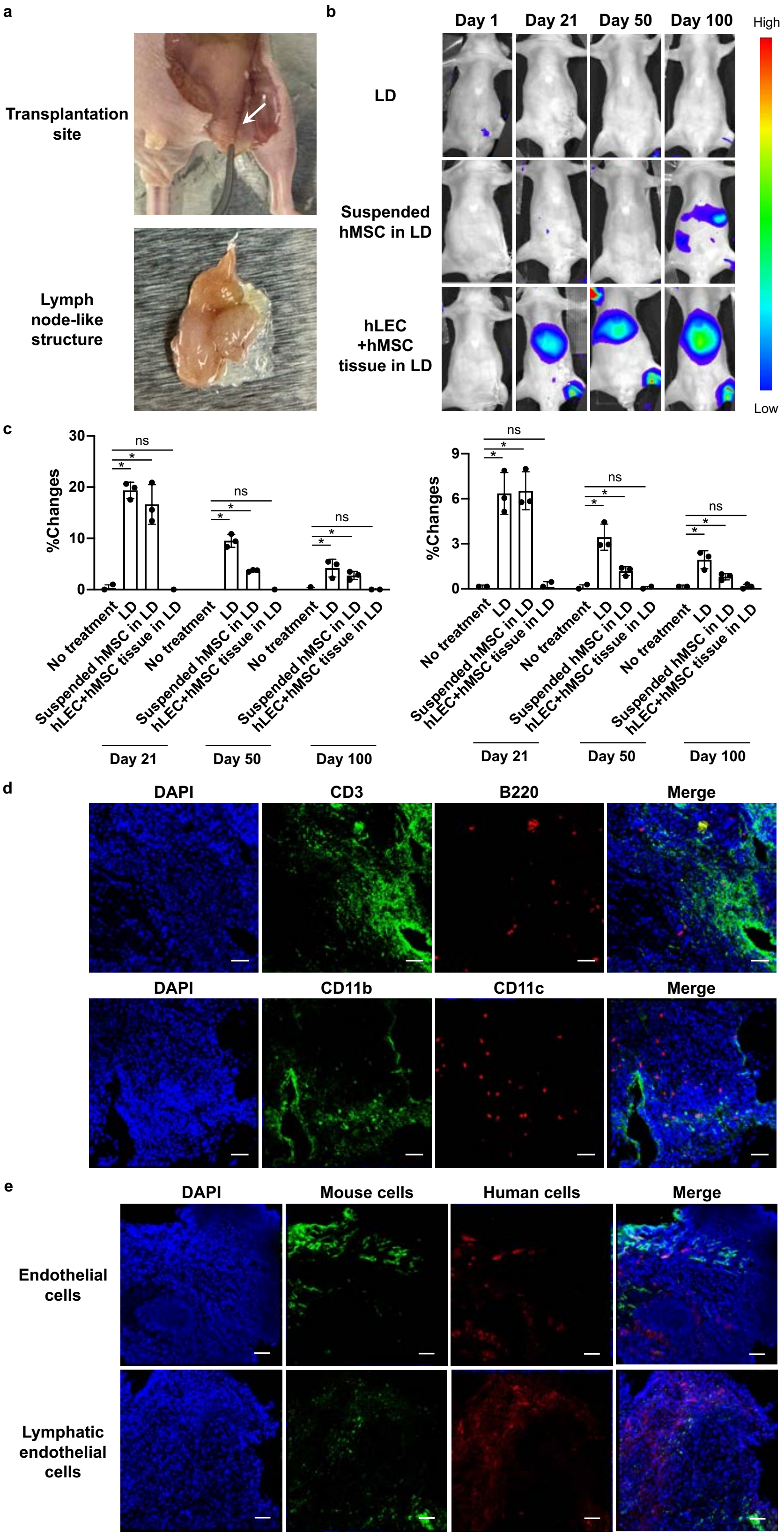
a
Transplantation site
Lymph node-like structure
b
Day 1
Day 21
Day 50
Day 100
High
Low
LD
Suspended hMSC in LD
hLEC +hMSC tissue in LD
c
%Changes
ns
No treatment
LD
Suspended hMSC in LD
hLEC+hMSC tissue in LD
Day 21
Day 50
Day 100
d
DAPI
CD3
B220
Merge
CD11b
CD11c
e
Mouse cells
Human cells
Endothelial cells
Lymphatic endothelial cells

256

257

Supplementary Fig. 13. | Sustained therapeutic effect in LD mice 100 days after transplantation of CeLyTs.

a, Typical images of the CeLyTs transplantation site in a right lower limb of an LD mouse (day 100) and the lymph node-like structure formed in hLEC+hMSC tissue-transplanted LD mice (day 100). Arrow, lymph node-like structure. **b**, *In vivo* fluorescence imaging after an injection of ICG to the footpad of right lower limbs of LD mice 1, 21, 50 and 100 days after the transplantation of suspended hMSC and hLEC+hMSC tissues. **c,** Size change of paws (left) and legs (right) in LD mice after transplantation of suspended hMSC and hLEC+hMSC tissues. The thickness of paws and legs was measured and the change rate for day 0 was calculated. Data represent the mean ± standard deviation from 3 independent experiments, and P-values were determined by two-sided Dunnett's test. $^*P < 0.05$ was considered statistically significant. ns, not significant. **d,** Representative immunofluorescence images of CD3 (a T cell marker), B220 (a B cell marker), CD11b (a macrophage marker) and CD11c (a dendritic cell marker) in lymph nodes or lymph node-like structures formed in hLEC+hMSC tissue-transplanted LD mice (day 100). Blue, DAPI; Green, CD3 or CD11b: Red, B220 or CD11c. Scale bars, 100 µm. **e**, Representative immunofluorescence staining for an endothelial marker CD31 and lymphatic endothelial cell marker Prox-1 in a lymph node-like structure formed in hLEC+hMSC tissue-transplanted LD mice (day 100). Blue, DAPI; Green, mouse-reactive CD31 or Prox-1: Red, human-reactive CD31 or Prox-1. Scale bars, 100 µm.
