# Supplementary material for: Reconstruction of the lymphatic system by transplantation of a centrifuge-based bioengineered lymphatic tissue
Source: Nat Commun. 2025 Nov 19;16:10154. doi: 10.1038/s41467-025-65121-3 (PMC12630873; doi:10.1038/s41467-025-65121-3)
Supplement: Supplementary file 2 — Reporting Summary [file 41467_2025_65121_MOESM2_ESM.pdf]

nature portfolio

Corresponding author(s): Kosuke Kusamori

Last updated by author(s): Oct 1, 2025

Reporting Summary

Nature Portfolio wishes to improve the reproducibility of the work that we publish. This form provides structure for consistency and transparency in reporting. For further information on Nature Portfolio policies, see our Editorial Policies and the Editorial Policy Checklist.

Statistics

For all statistical analyses, confirm that the following items are present in the figure legend, table legend, main text, or Methods section.

n/a	Confirmed	
☐	☒	The exact sample size (n) for each experimental group/condition, given as a discrete number and unit of measurement
☐	☒	A statement on whether measurements were taken from distinct samples or whether the same sample was measured repeatedly
☐	☒	The statistical test(s) used AND whether they are one- or two-sided<br>*Only common tests should be described solely by name; describe more complex techniques in the Methods section.*
☒	☐	A description of all covariates tested
☒	☐	A description of any assumptions or corrections, such as tests of normality and adjustment for multiple comparisons
☐	☒	A full description of the statistical parameters including central tendency (e.g. means) or other basic estimates (e.g. regression coefficient) AND variation (e.g. standard deviation) or associated estimates of uncertainty (e.g. confidence intervals)
☐	☒	For null hypothesis testing, the test statistic (e.g. F, t, r) with confidence intervals, effect sizes, degrees of freedom and P value noted<br>*Give P values as exact values whenever suitable.*
☒	☐	For Bayesian analysis, information on the choice of priors and Markov chain Monte Carlo settings
☒	☐	For hierarchical and complex designs, identification of the appropriate level for tests and full reporting of outcomes
☒	☐	Estimates of effect sizes (e.g. Cohen's d, Pearson's r), indicating how they were calculated

Our web collection on statistics for biologists contains articles on many of the points above.

Software and code

Policy information about availability of computer code

Data collection	Microscopy images were collected by BZ-X800 fluorescence microscope (Keyence, Osaka, Japan) and Leica SP8 laser scanning confocal microscope with LAS X Life Science software (Leica Microsystems, Wetzlar, Germany).<br>Relative light unit, fluorescence intensity and absorption intensity data were collected by 2104 EnVision multilabel plate reader (PerkinElmer, Waltham, MA, USA).<br>Imaging data were collected by In-Vivo Xtreme II (Bruker, Billerica, MA, USA) and IVIS Lumina III (PerkinElmer).<br>Flow Cytometry data were collected by BD FACSLyric (BD Biosciences Pharmingen, San Diego, CA, USA).<br>RT-qPCR data were collected by CFX Connect Real-Time PCR Detection System (Bio-Rad Laboratories, Hercules, CA, USA).
Data analysis	Flow Cytometry data were analyzed by FlowJo (BD Biosciences Pharmingen, v10.7.2).<br>Statistics were performed by JMP Pro (Jmp Statistical Discovery Llc, Tokyo, Japan, v17).<br>The intensity of the region of interest (ROI) signal at the transplantation site was quantified using Bruker Molecular Imaging software (Bruker).

For manuscripts utilizing custom algorithms or software that are central to the research but not yet described in published literature, software must be made available to editors and reviewers. We strongly encourage code deposition in a community repository (e.g. GitHub). See the Nature Portfolio guidelines for submitting code & software for further information.

Data

Policy information about availability of data

All manuscripts must include a data availability statement. This statement should provide the following information, where applicable:

- Accession codes, unique identifiers, or web links for publicly available datasets
- A description of any restrictions on data availability
- For clinical datasets or third party data, please ensure that the statement adheres to our policy

The data generated in this study are provided in the Source Data file included with this paper. No raw imaging data have been deposited in a public repository; access to the raw imaging data is restricted due to file size and data management constraints. Requests for these data can be directed to the corresponding authors, and access will be provided for academic research purposes within 4 weeks of the request. The raw numerical data supporting the findings of this study are included in the Source Data file.

Research involving human participants, their data, or biological material

Policy information about studies with human participants or human data. See also policy information about sex, gender (identity/presentation), and sexual orientation and race, ethnicity and racism.

Reporting on sex and gender	Cells used in this study are human primary adipose-derived stem cells (hMSCs) from Rohto Pharmaceutical Co., Ltd. (Osaka, Japan) and human primary lymphatic endothelial cells (hLECs) purchased from Takara Bio Inc. (Tokyo, Japan, C-12216, lot: 467Z001.2).
Reporting on race, ethnicity, or other socially relevant groupings	The hMSCs were isolated from adipose tissue from a single Japanese donor. The hLECs were isolated from juvenile foreskin (different locations) from a single caucasian (Donar age/Sex: 2/male) donor.
Population characteristics	N/A
Recruitment	N/A
Ethics oversight	N/A

Note that full information on the approval of the study protocol must also be provided in the manuscript.

Field-specific reporting

Please select the one below that is the best fit for your research. If you are not sure, read the appropriate sections before making your selection.

- [x] Life sciences
- [ ] Behavioural & social sciences
- [ ] Ecological, evolutionary & environmental sciences

For a reference copy of the document with all sections, see nature.com/documents/nr-reporting-summary-flat.pdf

Life sciences study design

All studies must disclose on these points even when the disclosure is negative.

Sample size	Results showed that at least 3 samples per group were needed to reveal the differences. The sample sizes were determined to be sufficient to perform statistical analyses.
Data exclusions	No data was excluded.
Replication	All attempts at replication were successful.
Randomization	Allocation was random.
Blinding	The investigations were blinded to group allocation during data collection and analysis.

Reporting for specific materials, systems and methods

We require information from authors about some types of materials, experimental systems and methods used in many studies. Here, indicate whether each material, system or method listed is relevant to your study. If you are not sure if a list item applies to your research, read the appropriate section before selecting a response.

Materials & experimental systems

n/a	Involved in the study	
	x	Antibodies
	x	Eukaryotic cell lines
x		Palaeontology and archaeology
	x	Animals and other organisms
x		Clinical data
x		Dual use research of concern
x		Plants

Methods

n/a	Involved in the study	
x		ChIP-seq
	x	Flow cytometry
x		MRI-based neuroimaging

Antibodies

Antibodies used

The primary antibody: 1:200 PROX-1 (Proteintech, Rosemont, IL, USA, 11067-2-AP), 1:500 mouse PROX-1 (Abcam, Cambridge, UK, ab101851), 1:500 human PROX-1 (Bio-Techne, Minneapolis, MN, USA, AF2727), 1:500 CD31 (Bio-Techne, NB600-1475), 1:500 mouse CD31 (Abcam, ab256569), 1:500 human CD31 (Abcam, ab76533), 1:100 B220/CD45R (Bio-Techne, NBP2-53303), 1:100 CD3 (Arigo, Zhubei, Taiwan, ARG22819), 1:50 CD11b (Abcam ab8878), 1:50 CD11c (ThermoFisher Scientific, Waltham, MA, USA, PA5-90208), 1:400 LYVE-1 (Thermo Fisher Scientific, BS-1311R), 1:500 Collagen Type I (Proteintech, 14695-1-AP), 1:50 HuNuC (Sigma-Aldrich, St. Louis, MO, USA, MAB1281C3)
The secondary antibody: 1:500 anti-rabbit Alexa488 (Thermo Fisher Scientific, A21206), 1:500 anti-rat Alexa488 (Thermo Fisher Scientific, A-11006), 1:500 anti-rabbit Alexa647 (Thermo Fisher Scientific, A31573), and 1:500 anti-goat Alexa647 (Thermo Fisher Scientific, A21447).

Validation

All primary antibodies, commercially available and are validated by suppliers as follows.
PROX-1
https://www.ptglab.co.jp/products/PROX1-Antibody-11067-2-AP.htm
mouse PROX-1
https://www.abcam.co.jp/products/primary-antibodies/prox1-antibody-bsa-and-azide-free-ab101851.html
human PROX-1
https://www.bio-techne.com/p/antibodies/human-prox1-antibody_af2727
CD31
https://www.bio-techne.com/p/antibodies/cd31-pecam-1-antibody-mec133_nb600-1475pe
mouse CD31
https://www.abcam.co.jp/products/primary-antibodies/cd31-antibody-mec-133-ab256569.html
human CD31
https://www.abcam.co.jp/products/primary-antibodies/cd31-antibody-epr3094-ab76533.html
B220/CD45R
https://www.novusbio.com/products/b220-cd45r-antibody-ptprc-1783r_nbp2-53303?srsltid=AfmBOoqnWen2TPxiaKWc7qW3cWn-YMzFzWRv0VY2RiIVZbD_2Vz_TjPV
CD3
https://www.arigobio.com/anti-CD3-epsilon-antibody-CD3-12-ARG22819.html
CD11b
https://www.abcam.co.jp/products/primary-antibodies/cd11b-antibody-m170-ab8878.html
CD11c
https://www.thermofisher.com/antibody/product/CD11c-Antibody-Polyclonal/PA5-90208
LYVE-1
https://www.thermofisher.com/antibody/product/LYVE-1-Antibody-Polyclonal/BS-1311R
Collagen Type I
https://www.ptglab.co.jp/products/COL1A2-Antibody-14695-1-AP.htm
HuNuC
https://www.merckmillipore.com/JP/ja/product/Anti-Nuclei-Antibody-clone-235-1-Cy3-conjugate,MM_NF-MAB1281C3

Eukaryotic cell lines

Policy information about cell lines and Sex and Gender in Research

Cell line source(s)	C3H10T1/2 cell line, NIH3T3 cell line
Authentication	C3H10T1/2 cell line was kindly provided by Dr. Hiroki Kagawa (Kyoto, Japan). NIH3T3 cell line was obtained from RIKEN Bioresource Center (RIKEN BRC) (Ibaraki, Japan).
Mycoplasma contamination	All cell line tested negative for mycoplasma contamination.
Commonly misidentified lines (See ICLAC register)	N/A

Animals and other research organisms

Policy information about studies involving animals; ARRIVE guidelines recommended for reporting animal research, and Sex and Gender in Research

Laboratory animals	Male nude BALB/c Slc-nu/nu mice (5–8-week old) were purchased from Sankyo Labo Service Co., Inc. (Tokyo, Japan)
Wild animals	The study did not involve wild animals.
Reporting on sex	In order to avoid experimental differences caused by animal sex, animals of the same sex were used in the same experiment.
Field-collected samples	The study did not involve field-collected samples.
Ethics oversight	All animal experimentations were conducted in accordance with the Institutional Animal Experimentation Committee of the Tokyo University of Science (latest approval number: Y23004).

Note that full information on the approval of the study protocol must also be provided in the manuscript.

Plants

Seed stocks	N/A
Novel plant genotypes	N/A
Authentication	N/A

Flow Cytometry

Plots

Confirm that:

- [x] The axis labels state the marker and fluorochrome used (e.g. CD4-FITC).
- [x] The axis scales are clearly visible. Include numbers along axes only for bottom left plot of group (a 'group' is an analysis of identical markers).
- [x] All plots are contour plots with outliers or pseudocolor plots.
- [x] A numerical value for number of cells or percentage (with statistics) is provided.

Methodology

Sample preparation	The samples were enzymatically digested with TrypLE™ Express (ThermoFisher Scientific, Waltham, MA, USA). After dissociation, cell suspensions were passed through a 70 µm cell strainer.
Instrument	BD FACSLyric
Software	BD FACSuite™ (v1.4.1), FlowJo™ (v10.7.2)
Cell population abundance	Sufficient number of cells were used in each experiment.
Gating strategy	Cells were defined based on Forward (FSC-A) and Side Scatter (SSC-A) parameters. Gating strategies were not performed in the study.

- [ ] Tick this box to confirm that a figure exemplifying the gating strategy is provided in the Supplementary Information.
